# Exploring the potential hazard of *Mycobacterium avium* subspecies *paratuberculosis* as a cause for Crohn’s disease

**DOI:** 10.14202/vetworld.2017.457-460

**Published:** 2017-04-28

**Authors:** Sukumar Bharathy, Lakshmanasami Gunaseelan, Kannan Porteen

**Affiliations:** Department of Veterinary Public Health and Epidemiology, Madras Veterinary College, Chennai - 600 007, Tamil Nadu, India

**Keywords:** Crohn’s disease, excretion, food chain, IS*900* polymerase chain reaction, *Mycobacterium avium* subspecies *paratuberculosis*, raw milk

## Abstract

**Background:**

*Mycobacterium avium* subspecies *paratuberculosis* (MAP) is the causative agent of Johne’s disease (JD), or paratuberculosis in ruminants has been suspected to be implicated in the pathogenesis of Crohn’s disease (CD) in humans with chronic inflammatory intestinal changes. As the hypothesis is now fast being recognized that MAP could possibly be the etiological agent of CD which is found to be excreted in milk of dairy animals subclinically or terminally ill with JD.

**Aim:**

The present study was aimed to detect MAP in milk by polymerase chain reaction (PCR) targeting IS*900* and to describe the excretion pattern of MAP in milk from asymptomatic lactating cows and does with relevance to the public health significance.

**Materials and Methods:**

A total of 77 milk samples were collected randomly from lactating animals which include cows (45) and does (32). All the 77 milk samples were processed to identify the presence of MAP by employing the direct IS*900* PCR as per the standard protocol.

**Results:**

Out of 77 milk samples from asymptomatic lactating animals, 12 (15.58%) were showed positivity for IS*900* PCR in which 5 (11.11%) were from lactating cows and 7 (21.87%) were from lactating does.

**Conclusion:**

In our study, 15.58% of milk samples showed IS*900* positivity which indicates the presence of subclinical MAP infection in lactating animals. Hence, there is a possibility for excretion of MAP through milk which can be a potential threat for CD in humans by raw milk consumption. Therefore, the prevention of MAP in the food chain need to be assured by sourcing raw products from animal herds free of MAP infection.

## Introduction

Crohn’s disease (CD) is one of a group of inflammatory bowel diseases that also includes ulcerative colitis. CD was first described in 1932 as a chronic inflammation of the terminal ileum [1]. In some areas in the USA in recent years, the incidence of CD is around 7-8 per 10^5^ populations per year [2]. No convincing explanation for the pathogenesis of CD currently exists; however, various environmental factors (e.g., pathogenic or non-pathogenic microbes, lifestyle, hygiene factors, diet, and stress) have been suggested as a cause for CD [3,4]. *Mycobacterium avium* subspecies *paratuberculosis* (MAP) is suspected as one of the causes for CD. MAP causes a disease in dairy cows and other animals that are similar to CD, called Johne’s disease (JD) or paratuberculosis [5], and MAP has been isolated in intestinal tissues of CD patients, thus giving rise to a hypothesis of there being a possible link between these two diseases [6]. Several studies have reported contrasting observations regarding the association of MAP and CD in humans. India has been an endemic area for intestinal tuberculosis in humans as well as JD in ruminants. Conventionally, India has been considered as a low prevalence area for CD. However, in the last decade, there has been a rapid increase in the prevalence of inflammatory bowel disease in India and other Asian countries [7].

MAP is shed intermittently by subclinically and clinically affected animals into feces and milk and colostrum and viable MAP has been also isolated from such animals [8]. MAP isolation from milk was first reported in 1935, in association with advanced clinical paratuberculosis. More recent studies have found MAP isolation rates in milk of up to 28% in clinically affected cows from State of Pernambuco, Brazil [9] and 48% in colostrums from the dam-daughter pairs given birth/being born on eight commercial dairy farms with endemic paratuberculosis located in Friesland [10] and 12% in milk of subclinical cases of bovines from Pennsylvania [11], with MAP DNA detection in pasteurized milk samples purchased from retail markets [12]. At present, it is not known whether commercial pasteurization effectively kills MAP in contaminated raw milk [12]. MAP has been identified in milk samples of dairy lactating animals with a history of JD by nested IS*900* polymerase chain reaction (PCR) and this bacterium can survive pasteurization [13]. A link between MAP, CD, and consumption of dairy products from raw milk is still to be demonstrated [14]. PCR is a rapid and sensitive method for the detection of MAP in milk and other types of samples [15].

Therefore, the study was aimed to detect MAP in milk by PCR targeting IS*900* and to describe the excretion pattern of MAP in milk from asymptomatic lactating cows and goats with relevance to the public health significance.

## Materials and Methods

### Ethical approval

There were no live animals used in this study; hence, there is no ethical approval necessary.

### Study population

A total of 77 milk samples were collected from organized dairy farms and the Madras Veterinary College (MVC) Teaching Hospital, Chennai, which included 45 lactating cow and 32 lactating does. None of these animals had been systematically examined for paratuberculosis by serology or fecal culture.

### Milk sampling

Before collection of milk, the teats were thoughtfully cleaned with warm water to avoid sample contamination from skin and feces. Milk samples (5-10 ml) were collected in a 10 ml (with V-shaped bottom) sterile graduated tube from all the quarters of each lactating animals (cows and does) by hand milking, discarded the first 1-2 ml and the samples were transported to laboratory of the Department of Veterinary Public Health and Epidemiology, MVC, Chennai, and refrigerated at 4°C until analysis, i.e., within 24 h from collection.

### Sample processing for DNA extraction

The milk samples were centrifuged at 3000 rpm for 15 min and the supernatant, including the hardened fat cream layer, was aspirated and discarded, except pellet. The resultant pellet was washed thrice in phosphate-buffered saline (pH 7.3) and centrifuged at 1000 rpm for 10 min. DNA was extracted from the pellet using commercial kit (DNA extraction kit, Qiagen, Hilden, Germany). The final product was stored at −20°C for subsequent PCR.

### PCR

IS*900* PCR was performed as per the procedure described by Pillai and Jayarao [16] using two primers. 5’primer sequence was 5’-CCGCTAATTGAGAGATGCGATTGG-3’ and the 3’ primer sequence was 5’AATCAA CTCCAGCAGCAGCGCGGCCTCG-3’ for detection of insertion sequence of 900 (IS*900*) of MAP which amplifies a 229 bp fragment. PCR reaction volume was set to 25 µl, 12.5 µl ×2 Master Mix (Ampliqon, Denmark), 1 µl of both forward and reverse primer (10 pmole/µl), and 4 µl DNA template and remaining volume where adjusted with nuclease free water. Thermal cycling was performed in a Eppendorf thermal cycler (Hamburg, Germany) and cycling conditions were as follows, initial denaturation at 94°C for 3 min, followed by 35 cycles for denaturation at 94°C for 1 min, annealing of primers at 55°C for 1 min, extension at 72°C for 1 min, and final extension at 72°C for 7 min.

### Agarose gel electrophoresis

The resulting PCR amplified products were electrophoresed in 1.5% agarose using sodium borate buffer (pH 8.2) (Fisher Scientific, India) with constant voltage of 100 V for 2 h. The DNA fragments were stained with ethidium bromide (1 mg/ml) and were visualized using ultraviolet-transilluminator. The size of the amplified product was checked by molecular weight DNA marker, 100 bp ladder (GeNei™, Bengaluru). The presence or absence of a 229 bp fragment was recorded.

## Results

**Figure-1:**
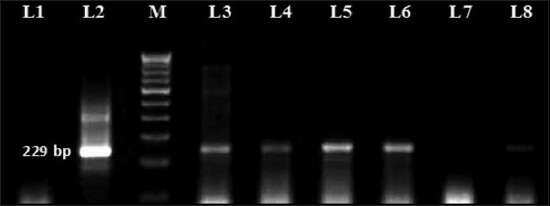
*Mycobacterium avium* subspecies *paratuberculosis*-specific amplicons (229 bp) using IS*900* specific primers in goat milk samples. M: Molecular weight DNA marker, 100 bp ladder; Lane 1: Negative control; Lane 2: Positive control; Lane 3,4,5,6 and 8: DNA test samples.

**Figure-2:**
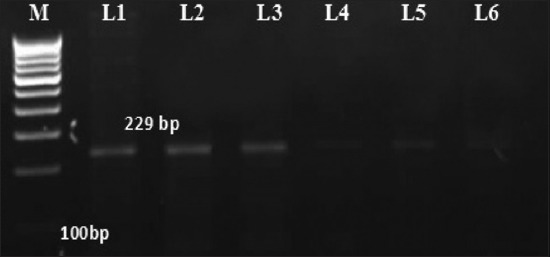
*Mycobacterium avium* subspecies *paratuberculosis* specific amplicons (229 bp) using IS*900* specific primers in cow milk samples. M: Molecular weight DNA marker, 100 bp ladder; Lanes 1-6: DNA test samples.

Out of 77 milk samples collected from asymptomatic lactating dairy cows and does, 12 (15.58%) were IS*900* PCR-positive, which included 5 (11.11%) lactating cows and 7 (21.87%) lactating does. Agarose gel analysis of the amplified products size of 229 bp considered as positive (Figures-1 and 2). The PCR products, however, differed in intensity. The differences in the intensity of the PCR-amplicons may be due to different concentrations of MAP in the randomly collected milk samples. This observation employing the most sensitive and rapid PCR-based test is clear evidence of MAP excretion in subclinically infected animals and offers the potential for spread to suckling young and possibly humans consuming raw milk.

## Discussion

This study was undertaken to prove the excretion of MAP in milk from lactating asymptomatic animals by PCR targeting IS*900* gene and to correlate the health risk associated with the consumption of milk and milk products from these animals. The most important routes of access of MAP to the food chain appear to be contaminated milk and milk products from infected ruminants [15]. Results of our study showed 15.58% positivity by IS*900* PCR indicated an evidence regarding the excretion of MAP organisms in milk and helped us to identify the subclinical carriers in general population of animals. In the past decade, several methods for the detection, isolation, and identification of MAP in milk have been described and most frequent method of MAP detection in milk is PCR [17]. The insertion element IS*900* is the mostly used target for identification and also differentiation of MAP from other mycobacteria [18]. In a similar fashion, many studies have been conducted utilizing IS*900* PCR to screen MAP organisms from bovine milk samples showed 3.6% (8/222) [19], 3.4% (29/851) positive [20], 7.8% [21], and 19.7% [22]. We have conducted similar kind of study in milk samples from 15 JD positive goats to establish the usefulness of milk as a non-invasive sample and we have detected MAP DNA in all 15 milk samples (100%), four nucleotide sequences were deposited in the GenBank with accession numbers of KJ882900 to KJ882903 [17]. Variation of results between researchers worldwide may be attributed to the fact that the samples were collected from the general population including the healthy animals, further the shedding of MAP organisms at detectable levels in milk is irregular and intermittent [23] and fecal contamination during sampling or udder invasion through the teat channel or as a result of systemic dissemination [24,25]. In our study, the detection of positives was higher hinting an obviously endemic environment since raw milk may also be contaminated with feces during the milking process. The feces can contain at least 10^6^ colony forming units per gram, indicating that fecal contamination of milk may be a critical point at which MAP can enter the food supply [26].

In addition, many reports worldwide has shown that dairy products are made from raw milk or milk that has been subjected to heating treatments less severe than those used for pasteurization [27-29]. Current commercial pasteurization standards may reduce number of viable MAP but do not ensure destruction of all bacilli [30]. A study by Stabel and Lambertz [12] demonstrated that MAP present in raw milk and can survive subpasteurization heating treatments; In India, Shankar *et al*. [31] reported a high presence of MAP in pooled raw milk (44%), commercially pasteurized milk (67%), and milk products (56%) indicating the survivability of these organisms even after heat treatment and risk associated with the consumption of milk and milk products from infected animals.

This study attempted to establish a causal relationship between MAP and CD by identifying the possible potential hazard of transmission through milk. Consumption of unpasteurized milk or milk products represents a low proportion of total milk intake; therefore, the extent raw milk could expose a population to MAP is limited. However, it still can be hypothesized that unpasteurized products have a greater amount of viable MAP than pasteurized products, and hence, the practice of consuming unpasteurized milk products may increase the exposure of a person to MAP, which may increase the risk of that person developing CD.

## Conclusion

Routine screening of animals for MAP by utilizing non-invasive milk sample will help us to identify subclinical carriers and major route of transmission of the disease through milk can be prevented by adopting appropriate methods such as proper pasteurization of milk and milk products. Results of our study clearly proved the excretion of MAP organisms in milk which needs further investigation to elucidate the role of MAP as a potential threat to humans and to devise strict biosecurity measures to combat the disease in both animals and humans.

## Authors’ Contributions

SB carried out sample collection, sample processing, and drafting the manuscript. LG supervised the research work. KP provided guidance for the research work and revised the manuscript. All authors read and approved the final manuscript.
